# Nomen est omen? How and when company name fluency affects return expectations

**DOI:** 10.1371/journal.pone.0287995

**Published:** 2023-08-16

**Authors:** Achiel Fenneman, Dirk-Jan Janssen, Sven Nolte, Stefan Zeisberger

**Affiliations:** 1 Department of Cognition, Emotion, and Methods in Psychology, University of Vienna, Vienna, Austria; 2 Institute for Management Research, Radboud University, Nijmegen, The Netherlands; 3 Department of Banking and Finance, University of Zurich, Zurich, Switzerland; University of Durham: Durham University, UNITED KINGDOM

## Abstract

Investors perceive stocks of companies with fluent names as more profitable. This perception may result from two different channels: a direct, non-deliberate affect toward fluent names or a deliberate interpretation of fluent names as a signal for company quality. We use preregistered experiments to disentangle these channels and test their limitations. Our results indicate the existence of a significant non-deliberate fluency effect, while the deliberate fluency effect can be activated and deactivated in boundary cases. Both effects are consistent across different groups of participants. However, whereas the fluency effect is strong in isolation, it has limitations when investors are confronted with additional information about the stock.

## 1 Introduction

When making investment decisions, investors can use a plethora of information. This ranges from technical information like stock returns, risk characteristics, and data derived from balance sheets and income statements to simple cues like the company name or ticker symbol. However, due to constraints in both time and cognitive resources, most investors are limited in their ability to properly incorporate all this information in their decisions, leading them to resort to strategies flowing from heuristic simplification [1].

One potential simplification may be centered around the *fluency* of an informational cue. Specifically, the fluency with which information can be processed has been found to affect human attitudes and judgements [2], with increased fluency generally resulting in feelings of positive affect [3] compared with information that is processed with low fluency. This in turn leads to a ‘fluency effect’ that can steer decision making both directly, that is, the fluency of an informational cue itself serves as a cue, as well as indirectly, that is, more fluent cues receive more weight in the decision problem at hand [4].

Our paper explores the channels, strength and robustness of the fluency effect in financial decision making, using a replication and extension of the experimental setting by [5]. Although [5] establish that return expectations are positively correlated with the fluency of a company’s name, their experiment does not (1) clarify the channel through which these expectations are affected, and (2), does not address the robustness of this result to the inclusion of additional information beyond the company name. In our paper we address these two issues.

Specifically, regarding the first issue, we argue that return expectations in [5] can be affected by two possible channels: (1) a fluency effect through affect toward fluent names, relating to a non-deliberate fluency effect, and (2) a deliberate consideration of name fluency as a signal for company performance. In the first case, investors simply have a naive preference for fluent company names based on affect. In the second case, investors believe that the name itself is a signal for company quality and financial performance, which leads them to make more optimistic return predictions for that company. The second channel therefore works through a deliberate consideration of the link between name fluency and company performance (e.g., better managers pick more fluent names) and not through affect—either directly or indirectly—toward the fluency of the company name itself.

The two channels—non-deliberate and deliberate—are immediately associated with the two ways that stimuli are processed: bottom-up and top-down [6]. Bottom-up processing happens fast, effortless, and automatic, while top-down processing is slow, requires mental effort, and is goal-driven. These channels are not mutually exclusive, but they constantly interact and provide feedback to each other [7]. Fluency effects can be classified according to which channel is dominant when processing a stimulus. There are other criteria to classify fluency effects [8], but the degree of deliberation proves especially useful in an economic setting: the processes are closely related to dual system theory [9], which has seen significant applications in economics and finance [10].

In a first step, we replicate the results of [5]: We observe higher return expectations for more fluently named stocks. Second, we demonstrate that this finding can not be exclusively explained as a function of experimenter demand. Third, we show that the effect persists even in an experimental condition in which participants are informed that company names are assigned to the companies at random. This excludes a deliberate interpretation of company names as signals for management quality and company performance. The persistence of the effect rules out the deliberate channel as the only channel through which fluency impacts return expectations and implies that investors exhibit a positive affect toward company names, lending support to a non-deliberate effect of fluency. However, in a condition where participants are told that a connection between company names and performance exists, fluency plays a significantly stronger role. This suggests that the deliberate effect can be ‘turned on’ through experimental manipulation. In our fourth and final experimental condition, we observe that the fluency effect disappears when participants are provided with additional (potentially relevant) information in the form of a price chart. This result offers an explanation for the findings from [11], who empirically demonstrate that stocks with fluent names exhibit higher returns as well as systematically undervalued prices. The explanation they put forth is that company names can indeed serve as signals for company quality, but naive investors fail to identify this. Our results support this explanation and showcase that, although investors perceive fluent company stocks as having superior future performance, this positive fluency effect is not strong enough to persist in conditions with additional stock (perceived) information. In turn, this might lead to the higher returns for fluently named stocks observed by [11].

The remainder of this paper is organized as follows: Section 2 provides an overview of the relevant literature, introduces our hypotheses and highlights the contribution, and Section 3 presents the preregistered experimental setup. Section 4 shows our results, while Section 5 provides a discussion of our findings and concludes the paper.

## 2 Related Literature, contribution and hypotheses

### 2.1 Literature review

The fluency of informational cues can differ due to a host of reasons, including print fonts [12], color contrast [13], a speaker’s accent [14], or repetition of the respective informational cue [15]. Generally, it is found that the more fluent a cue is perceived to be, the more positive affect it provokes, leading to more positive evaluations in the decision task at hand.

The existing literature offers several putative mechanisms to support such a direct positive effect of fluency. First, fluency can signal familiarity with the cue, implying that whatever is observed is unlikely to be harmful to the agent, leading to a positive response [16]. Second, fluency can induce positive affect as the brain relates the observed cue to the speed by which it can be successfully processed. When such progress is experienced as rewarding, then this relationship between the cue and its processability is translated into a positive affective response toward a fluent cue. This is especially true when the processability of the observed cue is larger than what would be expected based on the average experienced speed by which similar cues are processed [17].

The above suggests that perceptual fluency can be perceived as a cue that generally leads to a (more) positive evaluation of the observed information. For name fluency, this conclusion finds support in the literature. Specifically, name fluency affects (among others) trust and risk perception: [18] find that people invest more in trustees from an economic trust game when the name (either actual or assigned) of the trustee is more fluent. [19] present similar results and document that eBay sellers with relatively fluent usernames were rated as being more trustworthy than sellers with non-fluent usernames.

Given the strong link between name fluency and perceived risk and trustworthiness, it may not be surprising that fluency has also been found to influence decision making in the financial domain. [20] find that companies with fluent names have a higher breath of ownership (retail and mutual funds), higher average monthly turnover, and smaller transaction price impacts. [5] find that recently listed shares with fluent names, in both the form of its full name and in its ticker symbol, perform significantly better than stocks with non-fluent names, although the effect weakens over time. The authors hypothesize that this might be due to the release of more diagnostic information about the company over time, diminishing the relative effect of name fluency. [11] find that companies with fluent names have higher future profitability and systematically surprise analysts with unexpected positive earnings. This suggests that name fluency signals company quality. However, given their finding that stocks of companies with fluent names achieve higher abnormal returns than companies with non-fluent names, the informative value of fluent names seems only partially priced in, suggesting that investors do not incorporate name fluency to its full signaling potential.

One issue with empirical papers such as [20] or [11] is that they cannot completely isolate the channel through which name fluency might affect financial outcomes. Name fluency may stem from *non-deliberate* affect toward fluent names, but it could also arise from investors *deliberately* interpreting name fluency as a signal of management or company quality. Such a relation may be the result of better managers picking more fluent names for their companies, but also the result of more fluent names leading to better brand recognition and hence higher sales. One possible way to further explore this channel is via tailored experiments that allow some control over participants’ expectations and beliefs. However, the experimental literature on the relationship between name fluency and perception of stock characteristics is relatively scarce. One notable exception is the experiment in [5]. In their first experiment, participants were tasked with predicting the future performance of stocks, half of which were given fluent names and half of which were given non-fluent names. Their results show that participants expect fluent stocks to outperform non-fluent stocks and that fluent (non-fluent) stocks exhibit a positive (negative) expected absolute future return.

Although the experimental results from [5] lend support to a name fluency effect in financial decision making, that study did not focus on identifying the channel—non-deliberate or deliberate—through which company name fluency affects return expectations. Additionally, their experimental results are based on a relatively small sample (by today’s standards) of 29 participants. Moreover, the introduction to the experiment includes a description of the analyzed effect and places some focus on the role of the company name, potentially inducing experimenter demand. Given the interest and importance of understanding the real-world consequences of the effect [11, 20], we replicate and expand the [5] original study, using a substantially larger subject pool and a series of additional treatments. This allows us first to assess the robustness of the original findings and second to disentangle the non-deliberate and deliberate channels through which name fluency might affect return expectations. We describe further details of our experiment in Section 3.

### 2.2 Hypotheses

The central conjecture of this paper states that fluency of company names has a positive effect on return expectations. Based on the findings by [5], we predict that stocks with a more fluent name have a higher expected future performance than stocks with a non-fluent name. Therefore, our first hypothesis is formulated as:

*Hypothesis I: Return expectations are higher for stocks with fluent company names than for stocks with non-fluent names.*

Once this baseline result is established, we can move on to our goal of disentangling the potential drivers of this fluency effect; the non-deliberate affect channel and the deliberate channel. The non-deliberate channel functions via a “warm glow” toward the name. Investors feel positive (negative) affect towards fluent (non-fluent) names, and as a result expect higher (lower) returns of these companies. This channel is predicted to stem directly from the feelings experienced when confronted with the company name, regardless of any deliberate consideration by the investor. In contrast, the deliberate channel assumes that investors interpret company name fluency as a signal for company quality. Fluent (non-fluent) names indicate better (worse) company quality, resulting in higher (lower) expected returns. The exact reasoning may be diverse and differ between investors: examples could be better management quality (better managers choose better company names), higher sales through improved brand recognition, more frequent media coverage, or a broader investor base due to familiarity effects.

The non-deliberate fluency effect is defined as a bottom-up process: it is object-driven and happens fast, effortlessly, and automatically [21]. As such, the initial direction of the effect will be the same independent of additional information available to the observer [22]. While this non-deliberate, bottom-up fluency effect can be overruled, it requires mental effort to do so. The deliberate fluency effect, on the other hand, is defined as a top-down process: it is guided by deliberate thought of the observer and happens comparably slowly, requires mental effort, and is goal-driven [7]. Therefore, the direction of the deliberate fluency effect will be determined by information available to the observer. Any information about the link between a company’s name and its stock return will affect the deliberate fluency effect (as it changes the information available to the observer), but can not affect the non-deliberate fluency effect.

Both channels are not mutually exclusive. In fact, it is plausible that they are persistent and potentially reinforcing each other in the real world. As a consequence, it is difficult to isolate and test the two channels based on empirical data alone. In an experimental setting, however, it becomes possible to explore the boundaries of the effect. Whether or not the non-deliberate channel can be isolated is unclear based on the literature: making participants aware of the fluency effect might lead to an overall reduction of non-deliberate name fluency [8, 23]. However, [18, 19] show that (non-deliberate) name fluency persists even when participants have been made aware of its existence. Crucially, non-deliberate name fluency cannot be isolated from the name itself—as long as a company name is provided, the non-deliberate channel will be open and potentially affect return expectations. Therefore, we turn to analyzing the boundaries of the deliberate channel: for this channel, it is possible to vary the degree to which company names *can* act as signals for company quality. In this light, we formulate the second hypothesis as follows:

*Hypothesis IIa: The fluency effect becomes stronger when a stronger link between a company’s name and its stock performance can be expected.*
*Hypothesis IIb: The fluency effect becomes weaker when a weaker link between a company’s name and its stock performance can be expected.*

The non-deliberate effect should not be affected by variation in the link between a company’s name and its performance. Thus, a confirmation of both hypotheses would speak in favor of the existence of a deliberate fluency effect, even though it would not prove that it is the only channel in existence. In contrast, rejection of the hypotheses would support the non-deliberate effect and would challenge the existence of a deliberate channel.

Finally, we analyze the introduction of additional information. In particular, previous evidence suggests that the fluency effect is especially pronounced when judgments are more intuitive in nature and do not involve the active consideration of a cue’s informational content [24]. Still, the name fluency effect found in [19] persists even when participants are provided with objective information about seller reputation. Therefore, we test whether adding additional informational content to the decision framework reduces the fluency effect. There are at least two ways why additional information could impact the fluency effect. First, it could simply be a matter of attention. As more information pieces are available, less attention will be directed at each individual piece. This would affect both fluency channels. Second, additional information may be considered more relevant than the name, resulting in less weight put on the company name’s fluency in judging its performance. This way should only affect the deliberate fluency channel. However, both ways are likely to interact—more relevant information could draw more attention, and as a consequence remove attention from the company name. To explore this aspect further, we vary the relevance of the additional information in our experiment. We formulate our third and final hypothesis as:

*Hypothesis III: The fluency effect becomes weaker when additional information about the company is provided.*

## 3 Experimental setup

We conducted two experiments to test our hypotheses. The experimental setup is similar for both experiments and largely resembles that of [5]. After a short introduction and description of the experiment, participants see a list of 30 company names and are asked to state the expected future performance of each company’s stock over the next year. The names of these 30 companies are the 15 fluent and 15 non-fluent names used in the first experimental study by [5]. We had to exclude one of the non-fluent names from the original work (“Ulymnius”) in experiment 1 from further analysis due to a technical error in the programming of the experiment (the name did not show up correctly in the experiment). Since the experiment was conducted with non-native (but proficient) English speakers, we validated the categorization of company names in the fluent and non-fluent groups via a pretest. This pretest was conducted with 98 participants from the same population as the first experiment. Participants in this pilot study did not take part in our main experiment. In this pretest, we employ an online business name generator (https://namelix.com) to construct 120 random names. Participants in the pretest are asked to judge these 120 names in addition to the 30 names from [5] on their perceived degree of fluency. Specifically, we ask participants how easy it would be to pronounce the company name if they had to do so during a speech, on a scale from 0–100. The fluent (non-fluent) names from [5] ended up in the top (bottom) 25% of names, so they allow for a clear separation between fluent and non-fluent names in our sample. Therefore, we keep these names to make our setup as comparable as possible to the original study by [5], allowing for an exact replication. Fig 5 in the S1 Appendix visualizes the fluency ratings for the 30 companies.

### 3.1 Experiment 1

The first experiment (preregistration available at https://aspredicted.org/H1J_K2F) is a non-incentivized online experiment with 653 second- and third-year undergraduate students of business administration and economics recruited at a large Dutch research university in exchange for a small course credit. Subjects were recruited in September 2020 and participated online with written consent. For the course credit, participants entered their student ID, which was stored separately from their answers and deleted after the study to prevent the possibility of identification. As there are no right or wrong answers, we refrained from paying a variable monetary incentive. Given that the study by [5] did not provide any monetary incentives, we also refrained from paying a fixed compensation. Approximately half of our sample states to have prior investment experience. 60% of participants are male. Each participant had to state their belief about the future stock return for each company on a scale from -40% to +40% with 10pp intervals. While being a relatively rough measure, this is in accordance with [5].

Participants are randomly assigned to one of four different conditions. We select the four conditions in our experiment to test Hypotheses I, IIb, and III, and to establish the robustness of our results. The baseline condition 1A (*N* = 149) is a direct replication of [5] and uses the same introduction as their work. In this condition, we do not provide participants with any information about the 29 companies other than their names. The introduction includes a description of the analyzed effect and explicitly emphasizes the role of the company name, potentially inducing experimenter demand. Specifically, the following section of the introduction may result in an experimenter demand effect: “*A recent study has shown that many everyday people buy shares with very little information about the company whose shares they are buying. This study is designed to determine whether people are able to accurately predict the success of a company on the share market, based only on the name of that company*.” The observation of increased return expectations for fluent compared with non-fluent company names in this condition could be driven by three different aspects: the wording of the introduction (experimenter demand effects), company names as signals of company quality and future returns, or affect toward fluent names, which drives behavior in the absence of more meaningful information. Conditions 1B–1D altered the baseline design in single relevant aspects to isolate the potential drivers of the fluency effect. In condition 1B (*N* = 139), we change the introduction, removing all background information that could potentially create an experimenter demand effect. In condition 1C (*N* = 185), we add the following sentence to the instructions: “All names are artificially created and assigned to the companies at random”. This information is supposed to reduce the deliberate fluency effect: interpretation of company names as signals for the quality of management and company performance should be invalid if names are randomly assigned. Condition 1D (*N* = 179) is identical to condition 1B, except for the inclusion of a one-year stock price chart randomly assigned to each company, allowing participants to make decisions based on something other than the company name alone. This condition serves as a test of the strength and robustness of the fluency effect even when additional information is available. All instructions and conditions are available in S1 Appendix, Section 1.

### 3.2 Experiment 2

The second experiment (preregistration available at https://aspredicted.org/Z2S_F9C) is also a non-incentivized online experiment, conducted with the next cohort of second- and third-year undergraduate students of business administration and economics recruited one year after the first experiment at the same university. Subjects were recruited in the same way as in the first experiment in September 2022. We removed 36 participants from the analyses that had previously participated in the first experiment. The 528 participants that we analyzed were 64.5% male, had a mean age of 20.1 years, and include 45.2% who state that they have prior investment experience. The study received a waiver from the ethical board of the University of Zurich.

The purpose of the second experiment is to explore the boundaries of the deliberate / non-deliberate fluency effect as well as the role of additional information in greater detail. In addition to a replication of the baseline condition (1B in experiment one, 2B in experiment 2, *N* = 92) of the previous experiment, we introduce four more conditions. All conditions can be seen in Table 1.

**Table 1:** Overview of conditions.

Condition	Description
1A	Direct replication of [5]
1B	As 1A but removing all background information that could potentially create an experimenter demand effect
1C	As 1B but adding a sentence that all names are artificially created and assigned to the companies at random
1D	As 1B but including of a one-year stock price chart randomly assigned to each company
2A	As 2B but providing information that name is a signal for quality (increase the deliberate fluency effect)
2B	As 1B (replication)
2C	As 2B but adding an explicit statement that there should not be any correlation between a company’s name and its performance
2D	As 2B but presenting a random number as company identifier
2E	As 2B but presenting additional potentially useful information in the form of earnings per share

This table presents the overview of conditions in Experiments 1 and 2.

In condition 2A (*N* = 121) we attempt to increase the deliberate fluency effect by providing the following information in the introduction: “Empirical research has shown that there is a positive correlation between how easy it is to pronounce the name of a company and that company’s performance. Therefore, a company’s name could be seen as a signal for its quality.” Explicitly mentioning the empirically established link between name fluency and company performance should activate the deliberate channel, and allows to directly test Hypothesis IIa. Participants in this condition should expect a strong link between company names and company performance.

In contrast, in condition 2C (*N* = 118) we try to diminish the deliberate channel even further than in condition 1C by adding the following statement: “All names are artificially created and assigned to the companies at random. Therefore, there should not be any correlation between a company’s name and its performance in this survey.” While there could arguably still be some uncontrollable mental contamination [25] through which the deliberate channel gets activated, it should be reduced to the lower boundary by this explicit statement. Thus, this condition allows us to test Hypothesis IIb.

Finally, we introduce two further conditions to deeper explore the role of additional information. Specifically, we investigate the relevance of this information to further differentiate between pure attention effects and active consideration of this new information. The price path we introduced in experiment 1 contains information that is directly relevant for the prediction of future stock performance, affecting the decision through attention and active consideration. In condition 2D (*N* = 84) we present a random number we call company identifier in addition to the company name. This number cannot provide any relevance for future company or stock performance, but may draw attention away from the name. Thus, it allows to isolate the pure attention effect. Finally, condition 2E (*N* = 113) adds earnings per share. While earnings per share cannot be related to future company returns directly, it seems intuitive that higher earnings should result in better future performance. This condition is therefore a middle-ground between the two extremes from the other information conditions. Taken together, conditions 2D and 2E allow testing of Hypothesis III, and provide a better understanding of the role of additional (perceived) information for fluency effects.

## 4 Results

For both experiments, we first test the effect of name fluency on return expectations. Since each participant saw a total number of 15 fluent and 15 non-fluent stocks, we compute the individual fluency-effect as the difference between expected returns of fluent and non-fluent company names. We analyze the differences between conditions based on these aggregated scores through a series of *t*-tests. All results are robust to running mixed effects OLS regressions instead. Results from these regressions are not reported here, but are available from the authors upon request.

**Fig 1:**
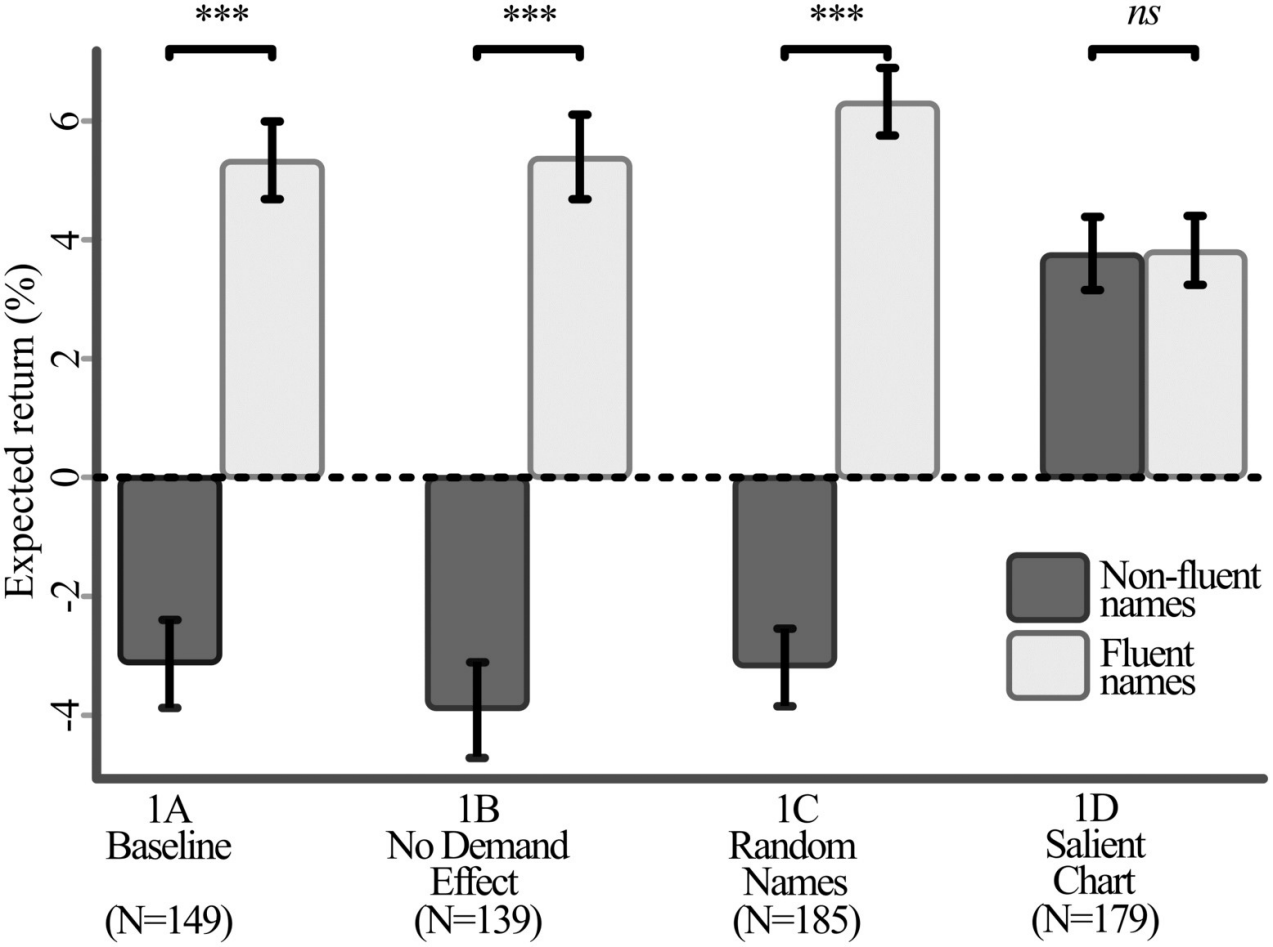
Average return expectations for fluent and non-fluent company names, by treatment of experiment 1. Error bars and significance based on within-subject paired *t*-tests.

In the first experiment, results show a significant difference between the expected returns of both the within-subject fluency treatment, as well as a significant between-subject effect of the experimental conditions. Table 2 provides summary statistics for the expected returns, outlined per fluent and non-fluent companies as well as comparisons between the conditions. These differences are graphically depicted in Fig 1.

**Table 2:** Fluency effect, experiment 1.

Cond.	*n*	Return expectations (fluent)	Standard deviation (fluent)	Return expectations (non-fluent)	Standard deviation (non-fluent)	Fluency effect	*t*	df	*p*
1A	149	5.34	6.31	-3.14	7.58	8.48	8.91	148	<.001
1B	139	5.51	7.27	-3.87	8.10	9.38	8.63	138	<.001
1C	185	6.32	7.33	-3.20	7.52	9.52	10.82	184	<.001
1D	179	3.85	6.39	3.82	6.69	0.03	0.08	178	0.938

The table reports average return expectations, separated by whether the company name is fluent or not. A positive difference between the two (i.e., higher return expectations for fluent companies) indicates a positive fluency effect. We test whether this fluency effect is significantly different from zero via within-subject, paired t-tests.

We observe a significant fluency effect for conditions 1A, 1B, and 1C, where return expectations for fluent stocks are significantly higher than for non-fluent stocks. In condition 1A, participants predict a mean return of +5.34% for companies with a fluent name, versus a mean return of –3.14% for companies with a non-fluent name (paired samples *t*[148] = 8.91, *p* <.001). In condition 1B, participants predict a mean return of +5.51% and –3.87% for fluent and non-fluent names (paired samples *t*[138] = 8.63, *p* <.001). In condition 1C, these mean predicted returns are +6.32% and -3.2% (paired samples *t*[184] = 10.82, *p* <.001). Notably, in all three conditions we observe a consistent sign change depending on the fluency category. Conversely, we do not observe a significant fluency effect in condition 1D, where participants state a mean predicted return of +3.85% and +3.82% for companies with fluent and non-fluent names, respectively (paired samples *t*[178] = 0.08, *p* = .938).

We next conduct a series of Welch-corrected independent-samples *t*-tests to verify whether the strength of the fluency effect differs between conditions (Table 3). We do not observe a significant difference in the strength between conditions 1A, 1B and 1C (all *p* >.05), whilst all three of these conditions significantly differ from condition 1D (all *p* <.001).

**Table 3:** Difference-in-difference tests, Experiment 1.

	Versus A				Versus B				Versus C			
Cond.	Diff	*t*	df	*p*	Diff	*t*	df	*p*	Diff	*t*	df	*p*
1B	0.90	0.62	278.29	.535								
1C	1.04	0.81	320.69	.421	0.15	0.1	286.13	.917				
1D	-8.45	-8.15	202.60	<.001	-9.34	-8.05	177.60	<.001	-9.49	-9.77	260.38	<.001

The table reports difference-in-difference tests, by condition. Specifically, the table reports results from between-subject t-tests of the average difference between return expectations of companies with fluent and non-fluent names (’fluency effect’). The fluency effect is calculated on a participant level.

Based on results from our first experiment, we can confirm Hypothesis I for all conditions but condition 1D, where we add additional information. Comparing conditions 1B and 1C, we observe no statistically significant difference. This insignificant difference complicates statements regarding the accuracy of Hypothesis IIb. We will address them further in the second experiment. Hypothesis III is confirmed by comparing condition 1D to the other conditions—the fluency effect is not only significantly weaker when additional information about the company is provided, it even completely vanishes in our experimental setup.

**Fig 2:**
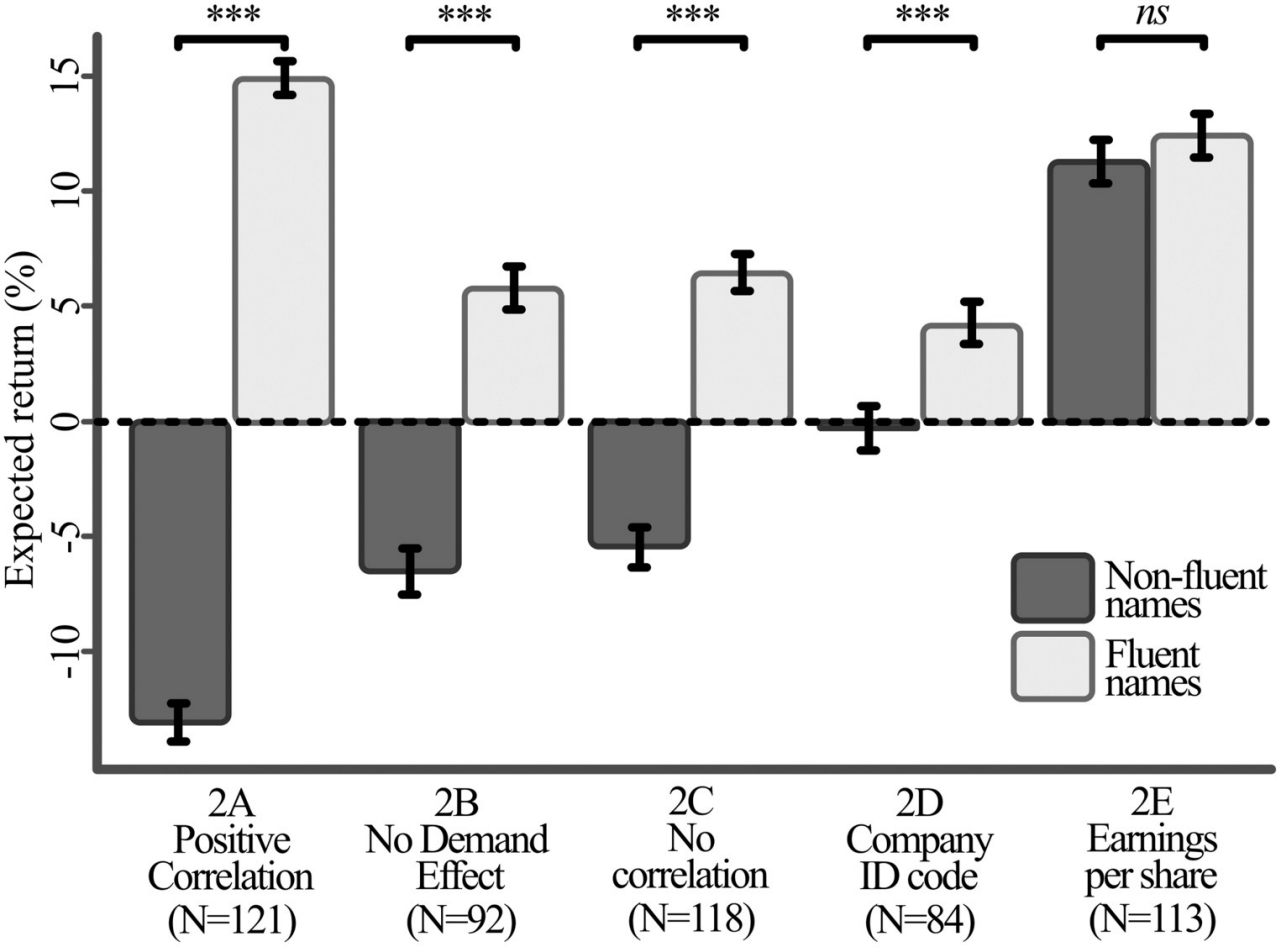
Average return expectations for fluent and non-fluent company names, by treatment of experiment 2. Error bars and significance based on within-subject paired *t*-tests.

Results from Experiment 2 support a significant difference between the expected returns of both the within-subject fluency treatment, as well as a significant between-subjects effect of the experimental conditions. Again, we collapse all fluency measurements to a per-participant level for post-hoc comparison via paired *t*-tests. Table 4 provides summary statistics for the expected returns, outlined per fluent and non-fluent companies as well as comparisons between the conditions. Results are visualized in Fig 2.

**Table 4:** Fluency effect, Experiment 2.

Cond.	*n*	Return expectations (fluent)	Standard deviation (fluent)	Return expectations (non-fluent)	Standard deviation (non-fluent)	Fluency effect	*t*	df	*p*
2A	121	14.91	15.48	-13.09	17.70	28.01	18.32	120	<.001
2B	92	5.79	17.47	-6.52	18.49	12.31	7.74	91	<.001
2C	118	6.46	16.57	-5.47	18.34	11.93	10.39	117	<.001
2D	84	4.28	16.37	-0.29	17.18	4.57	3.44	83	0.001
2E	113	12.41	19.44	11.29	19.87	1.13	1.42	112	0.159

The table reports average return expectations, separated by whether the company name is fluent or not. A positive difference between the two (i.e., higher return expectations for fluent companies) indicates a positive fluency effect. We test whether this fluency effect is significantly different from zero via within-subject, paired t-tests.

We observe a significant fluency effect for treatments 2A, 2B, 2C and 2D. As in the first experiment, this fluency effect is notably characterized by a difference in sign for the expected returns between companies with non-fluent versus fluent names. In condition 2A participants predict a mean return of +14.91% for fluent and -13.09% for non-fluent names (paired samples *t*[120] = 18.32, *p* <.001). In condition 2B, participants predict mean returns of +5.79% versus -6.52% (paired samples *t*[91] = 7.74, *p* <.001). In condition 2C participants predict mean returns of +6.46% versus -5.47% (paired samples *t*[117] = 10.39, *p* <.001), and in condition 2D participants predict mean returns of +4.38% versus -0.29% (paired sampled *t*[83] = 3.44, *p* = 0.001). We do not observe a significant fluency effect in condition 2E, with participants reporting mean expected returns of +12.41% for companies with fluent names, versus +11.29% for companies with non-fluent names (paired samples *t*[112] = 1.42, *p* = .159).

We next conduct a series of Welch-corrected independent-samples *t*-tests to verify whether the strength of the fluency effect differs between conditions (Table 5). The results of these difference-in-difference tests do not yield a significant difference in the strength of the fluency effect between conditions 2B and 2C (both *p* >.05). All other pair-wise comparisons result in significant differences in the strength of the fluency effect (all *p* <.05).

**Table 5:** Difference-in-difference tests, Experiment 2.

Cond.	Versus A				Versus B				Versus C				Versus D			
	Diff	t	df	p	Diff	t	df	p	Diff	t	df	p	Diff	t	df	p
2B	-15.69	-7.11	204.43	<.001												
2C	-16.08	-8.41	221.29	<.001	-0.39	-0.2	173.72	.845								
2D	-23.43	-11.56	202.59	<.001	-7.74	-3.73	171.05	<.001	-7.36	-4.19	181.27	<.001				
2E	-26.88	-15.59	179.67	<.001	-11.18	-6.29	135.29	<.001	-10.8	-7.73	206.61	<.001	-3.44	-2.22	139.72	.028

The table reports difference-in-difference tests, by condition. Specifically, the table reports results from between-subject t-tests of the average difference between return expectations of companies with fluent and non-fluent names (’fluency effect’). The fluency effect is calculated on a participant level.

We can confirm Hypothesis I for conditions 2A-2D of the second experiment—there is a significant fluency effect in all of these conditions. Experiment 2 also allows testing Hypotheses IIa and IIb more rigorously. If we take condition 2B as the baseline, we observe a significant increase in the fluency effect as we increase the (perceived) link between a company’s name and its performance. This confirms Hypothesis IIa. Hypothesis IIb, however, cannot be confirmed by our data—starting from the baseline condition 2B, we do not observe a significantly weaker fluency effect in condition 2C, where the link between a company’s name and its performance has explicitly been weakened. These results speak in favor of the existence of both fluency effects—deliberate and non-deliberate. The deliberate effect can be activated by explicitly stating a relation between name fluency and company performance. However, we did not manage to reduce the baseline fluency effect by explaining to participants that there cannot be any relation between fluency and stock performance. This failure to reduce the baseline fluency effect indicates the existence of a non-deliberate effect, which remains even in the boundary case where deliberate fluency considerations are weakened as much as possible. Finally, we can confirm Hypothesis III in our second experiment as well. The fluency effect is weaker for conditions 2D and 2E than for all other conditions. The result for condition 2D indicates that this perceived information effect is at least partially based on attention, since the company ID code is only a random number that cannot be related to company performance. The fact that the difference between conditions 2D and 2E is significant indicates that the information effect is also related to the quality of (perceived) information. It is sensible to assume that participants deem earnings per share to be more relevant for company performance than a random company ID. Thus, the relevance of additional (perceived) information seems to play a role.

## 5 Discussion

Previous empirical findings indicate that investment decisions and financial outcomes are affected by the fluency of company names: companies with a more perceptually fluent name have an increased investment propensity and firm value [20, 26]. However, the observational nature of these findings does not allow for a direct investigation of the channels that give rise to this name fluency effect. In this paper, we study the effect of fluency on investment decision making in a controlled laboratory environment, allowing us to disentangle the potential drivers of the fluency effect, and we thus provide important insights for the literature on name fluency in the financial domain.

Our experiments start with a replication of the original experiment of the first study of [5]. We confirm their findings in a larger sample. We additionally include multiple conditions to explore the driving forces behind the fluency effect in the context of return expectations. We observe the name fluency effect even in conditions where the potential effect of the deliberate channel is reduced to the lower boundary, speaking in favor of non-deliberate affect. At the same time, pushing the deliberate channel to its upper boundary results in a significantly stronger fluency effect, which indicates that the deliberate channel can at least be activated. The fluency effect is, yet, not sufficiently strong to persist in situations where additional (perceived) information is provided, augmenting the empirical evidence provided by [11].

Our experiment disentangles the immediate effect of fluency on return expectations through either direct, non-deliberate affect or deliberate interpretation as a signal. However, several more subtle channels that might impact the relationship between fluency and return expectations could be explored in future work using a similar experimental design. For example, as fluency and memory are highly correlated, company names that are more fluent are also likely to be more memorable. Memory quality, in turn, has an effect on belief formation [27] and decision making [28]. Therefore, fluency and investment behavior might show positive correlations in a setting where memory becomes important. Fluency might also play an indirect role through attention and preselection processes; investors often need to make some form of preselection before they start acquiring additional information about a stock [29]. If fluent company names draw attention, companies with fluent names may end up in the relevant choice set more often than non-fluent companies. Both of these indirect channels require a different experimental setup than the one currently employed. Our results and design can serve as a starting point for future research investigating these effects.

Our observed results could conceivably be driven by participants who either had previous experience in investing, and/or had a strong prior in favor of the fluency effect. To exclude these alternative interpretations, Experiment 2 includes two survey items (completed by participants consecutive to the main data collection) in which participants state whether they have any prior experience in investing, as well as whether they believe that the fluency of a company’s name can predict its future performance in the real world. To gain further insights into the drivers of the observed effects, we run an additional exploratory sub-sample analysis on four groups: participants who do or do not have any experience in investing, and participants who believe or do not believe in the predictive powers of name-fluency on company performance. The results of these questionnaire items are reported in the Appendix, Section 3. Overall, experience in investing does not yield a difference in observed fluency effects in any of the five conditions. This is true for the full sample and also for all five conditions separately. Hence, investment experience has no influence on the name-fluency effect in our setting. In contrast, participants who believe in the fluency effect display a larger effect in conditions 2A, 2C and 2D. Despite these differences, we observe a significant fluency effect in conditions 2A, 2B and 2C for all four subgroups, and hence even for the participants who claim to not believe in the name-fluency effect. This provides further evidence for the importance of the non-deliberate channel.

In conclusion, our results indicate that the fluency of a company’s name factors into financial decision making. We find that when only the company name is provided, the fluency of this name is an important cue in decision making. This even holds true when participants cannot use company names as signals for management quality and company performance. Finally, the observed fluency effect disappears with the inclusion of (potentially more meaningful) information. Therefore, our results suggest that affect toward fluency exists but might be outweighed by additional relevant perceived information about the performance of a company. This result relates to the empirical findings in [11, 20], that the fluency effect exists but the signaling quality of company names is not sufficiently accounted for by naive investors. The overall existence of the fluency effect can be explained by information overload—investors face an abundance of other informational sources in the real world, which may drive investors back to simplifying heuristics, such as a non-deliberate fluency effect. At the same time, this information overshadows the fluency effect to an extent that it is not sufficiently strong to fully account for the information contained in company name fluency.

## Supporting information

S1 Appendix (ZIP)

## References

[1] Hirshleifer D. Investor Psychology and Asset Pricing. The Journal of Finance. 2001;56(4):1533–1597. doi: 10.1111/0022-1082.00379

[2] Topolinski S. The sources of fluency: Identifying the underlying mechanisms of fluency effects. In: The experience of thinking: How the fluency of mental processes influences cognition and behaviour. New York, NY, US: Psychology Press; 2013. p. 33–49.

[3] Winkielman P, Cacioppo J. Mind at ease puts a smile on the face: Psychophysiological evidence that processing facilitation elicits positive affect. Journal of personality and social psychology. 2001;81:989–1000. doi: 10.1037/0022-3514.81.6.989 11761320

[4] Shah A, Oppenheimer D. Easy Does It: The Role of Fluency in Cue Weighting. Judgment and Decision Making. 2007;2:371–379.

[5] Alter AL, Oppenheimer DM. Predicting short-term stock fluctuations by using processing fluency. Proceedings of the National Academy of Sciences. 2006;103(24):9369–9372. doi: 10.1073/pnas.0601071103 PMC1482615 16754871

[6] Leynes PA, Addante RJ. Neurophysiological evidence that perceptions of fluency produce mere exposure effects. Cognitive, Affective, & Behavioral Neuroscience. 2016;16:754–767. doi: 10.3758/s13415-016-0428-1 27106854

[7] Gilbert CD, Sigman M. Brain states: top-down influences in sensory processing. Neuron. 2007;54(5):677–696. doi: 10.1016/j.neuron.2007.05.019 17553419

[8] Alter AL, Oppenheimer DM. Uniting the Tribes of Fluency to Form a Metacognitive Nation. Personality and Social Psychology Review. 2009;13(3):219–235. doi: 10.1177/1088868309341564 19638628

[9] Kahneman D. Thinking, fast and slow. New York: Farrar, Straus & Giroux; 2011.

[10] Shleifer A. Psychologists at the gate: a review of Daniel Kahneman’s thinking, fast and slow. Journal of Economic Literature. 2012;50(4):1080–1091. doi: 10.1257/jel.50.4.1080

[11] Montone M, van den Assem MJ, Zwinkels RC. Company Name Fluency and Stock Returns. Available at SSRN 3123201. 2022;.

[12] Song H, Schwarz N. If It’s Hard to Read, It’s Hard to Do: Processing Fluency Affects Effort Prediction and Motivation. Psychological Science. 2008;19(10):986–988. doi: 10.1111/j.1467-9280.2008.02189.x 19000208

[13] Silva RR, Garcia-Marques T, Mello J. The differential effects of fluency due to repetition and fluency due to color contrast on judgments of truth. Psychological research. 2016;80(5):821–837. doi: 10.1007/s00426-015-0692-7 26224218

[14] Dragojevic M, Giles H, Beck AC, Tatum NT. The fluency principle: Why foreign accent strength negatively biases language attitudes. Communication Monographs. 2017;84(3):385–405. doi: 10.1080/03637751.2017.1322213

[15] Feustel TC, Shiffrin RM, Salasoo A. Episodic and lexical contributions to the repetition effect in word identification. Journal of Experimental Psychology: General. 1983;112(3):309. doi: 10.1037/0096-3445.112.3.309 6225826

[16] Zajonc RB. Emotions. In: The handbook of social psychology, Vols. 1-2, 4th ed. New York, NY, US: McGraw-Hill; 1998. p. 591–632.

[17] Carver CS, Scheier MF. Origins and functions of positive and negative affect: A control-process view. Psychological Review. 1990;97(1):19–35. doi: 10.1037/0033-295X.97.1.19

[18] Zürn M, Topolinski S. When trust comes easy: Articulatory fluency increases transfers in the trust game. Journal of Economic Psychology. 2017;61:74–86. doi: 10.1016/j.joep.2017.02.016

[19] Silva RR, Unkelbach C. Fluent processing leads to positive stimulus evaluations even when base rates suggest negative evaluations. Consciousness and Cognition. 2021;96:103238. doi: 10.1016/j.concog.2021.103238 34784558

[20] Green TC, Jame R. Company name fluency, investor recognition, and firm value. Journal of Financial Economics. 2013;109(3):813–834. doi: 10.1016/j.jfineco.2013.04.007

[21] Reber R, Schwarz N, Winkielman P. Processing fluency and aesthetic pleasure: Is beauty in the perceiver’s processing experience? Personality and social psychology review. 2004;8(4):364–382. doi: 10.1207/s15327957pspr0804_3 15582859

[22] Forster M, Gerger G, Leder H. Everything’s relative? Relative differences in processing fluency and the effects on liking. Plos One. 2015;10(8):e0135944. doi: 10.1371/journal.pone.0135944 26288314 PMC4545584

[23] Schwarz N, Jalbert M, Noah T, Zhang L. Metacognitive experiences as information: Processing fluency in consumer judgment and decision making. Consumer Psychology Review. 2021;4(1):4–25. doi: 10.1002/arcp.1067

[24] Winkielman P, Schwarz N, Fazendeiro TA, Reber R. The hedonic marking of processing fluency: Implications for evaluative judgment. In Musch J. & Klauer K. C. (Eds.). In: The psychology of evaluation: Affective processes in cognition and emotion. Lawrence Erlbaum Associates Publisher; 2003. p. 189–217.

[25] Wilson TD, Brekke N. Mental contamination and mental correction: unwanted influences on judgments and evaluations. Psychological bulletin. 1994;116(1):117. doi: 10.1037/0033-2909.116.1.117 8078969

[26] Chan CSR, Park HD, Patel P. The effect of company name fluency on venture investment decisions and IPO underpricing. Venture Capital. 2018;20(1):1–26. doi: 10.1080/13691066.2017.1334369

[27] Enke B, Schwerter F, Zimmermann F. Associative Memory and Belief Formation. NBER Working Paper. 2020;.

[28] Bordalo P, Gennaioli N, Shleifer A. Memory, attention, and choice. The Quarterly journal of economics. 2017;.

[29] Agnew JR, Szykman LR. Asset allocation and information overload: The influence of information display, asset choice, and investor experience. The Journal of Behavioral Finance. 2005;6(2):57–70. doi: 10.1207/s15427579jpfm0602_2

