# Supplementary material for: Nomen est omen? How and when company name fluency affects return expectations
Source: PLoS One. 2023 Aug 16;18(8):e0287995. doi: 10.1371/journal.pone.0287995 (PMC10431612; doi:10.1371/journal.pone.0287995)
Supplement: S1 Appendix — (ZIP) [file pone.0287995.s001.zip › Appendix.pdf]

# Appendix to: Nomen est omen? How and when company name fluency affects return expectations

Achiel Fenneman[1,2], Dirk-Jan Janssen[1], Sven Nolte[1], Stefan Zeisberger[1,3] *

July 2, 2023

## 1 Introductions

These are the introductions used in our experiments, by treatment condition:

**Experiment 1, Condition A:**

A recent study has shown that many people buy shares with very little information about the company whose shares they are buying.

This study is designed to determine whether people are able to accurately predict the success of a company on the share market, based only on the name of that company.

Following is a list of 30 companies. Based on the name of each share alone, please indicate the extent to which you think this share will increase or decrease in value over the next 12 months, or whether you think its value will remain stable.

Please use the response scale on the following page when indicating your estimate of the share's performance.

*[1] Institute for Management Research, Radboud University, Nijmegen, The Netherlands. Corresponding author is sven.nolte@ru.nl, Heyendaalseweg 141, 6525 AJ Nijmegen, The Netherlands.
[2] Department of Cognition, Emotion, and Methods in Psychology, University of Vienna, Austria
[3] Department of Banking and Finance, University of Zurich, Switzerland

**Experiment 1, Conditions B and D and Experiment 2, Condition B:**

The following is a list of 30 companies. Please indicate for each share the extent to which you think this share will increase or decrease in value over the next 12 months, or whether you think its value will remain stable.

Please use the response scale on the following page when indicating your estimate of the share's performance.

**Experiment 1, Condition C:** The following is a list of 30 companies. Please indicate for each share the extent to which you think it will increase or decrease in value over the next 12 months, or whether you think its value will remain stable.

All names are artificially created and assigned to the companies at random.

Please use the response scale on the following page when indicating your estimate of the share's performance.

**Experiment 2, Condition C:** The following is a list of 30 companies. Please indicate for each share the extent to which you think it will increase or decrease in value over the next 12 months, or whether you think its value will remain stable.

All names are artificially created and assigned to the companies at random. Therefore, there should not be any correlation between a company's name and its performance in this survey.

Please use the response scale on the following page when indicating your estimate of the share's performance.

**Experiment 2, Condition D:** The following is a list of 30 companies with information about them. The last column contains the unique Company Identifier Code for each company.

Please indicate for each share the extent to which you think this share will increase or decrease in value over the next 12 months, or whether you think its value will remain stable.

Please use the response scale on the following page when indicating your estimate of the share's performance.

**Experiment 2, Condition E:** The following is a list of 30 companies with information about them. The last column contains the 12-month Earnings Per Share (EPS) for each company.

Please indicate for each share the extent to which you think this share will increase or decrease in value over the next 12 months, or whether you think its value will remain stable.

Please use the response scale on the following page when indicating your estimate of the share's performance.

## 2 Decision screens

Figures 1, 2, 3 and 4 present screenshots of the decisions in the experiment:

| Share Name | Estimated Performance over next 12 months | | | | | | | | |
|---|---|---|---|---|---|---|---|---|---|
| *Maerst* | -40% | -30% | -20% | -10% | 0% | +10% | +20% | +30% | +40% |
| *Undersill* | -40% | -30% | -20% | -10% | 0% | +10% | +20% | +30% | +40% |
| *Foleman* | -40% | -30% | -20% | -10% | 0% | +10% | +20% | +30% | +40% |
| *Kellings* | -40% | -30% | -20% | -10% | 0% | +10% | +20% | +30% | +40% |
| *Mayville* | -40% | -30% | -20% | -10% | 0% | +10% | +20% | +30% | +40% |
| *Vander* | -40% | -30% | -20% | -10% | 0% | +10% | +20% | +30% | +40% |
| *Adderley* | -40% | -30% | -20% | -10% | 0% | +10% | +20% | +30% | +40% |

Figure 1: Decision screen A-C, both experiments

| Share Name | Estimated Performance over next 12 months | Chart |
|---|---|---|
| ***Mayville*** | What performance do you expect from Mayville over the **next** 12 months? <br> -40% \| -30% \| -20% \| -10% \| 0% \| +10% \| +20% \| +30% \| +40% | 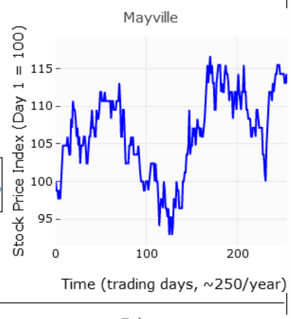  |
| ***Foleman*** | What performance do you expect from Foleman over the **next** 12 months? <br> -40% \| -30% \| -20% \| -10% \| 0% \| +10% \| +20% \| +30% \| +40% | 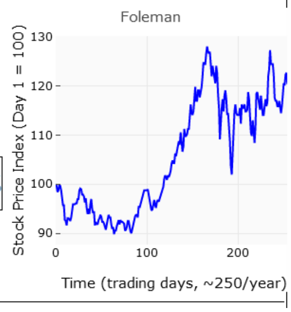  |

Figure 2: Decision screen D, Experiment 1

| Share | Estimated Performance over next 12 months | | | | | | | | | EPS (past 12 months) |
|---|---|---|---|---|---|---|---|---|---|---|
| ***Clearman*** | -40% | -30% | -20% | -10% | 0% | +10% | +20% | +30% | +40% | 5.91 |
| ***Fyndwyck*** | -40% | -30% | -20% | -10% | 0% | +10% | +20% | +30% | +40% | 7.41 |
| ***Mayville*** | -40% | -30% | -20% | -10% | 0% | +10% | +20% | +30% | +40% | 3.81 |
| ***Jillman*** | -40% | -30% | -20% | -10% | 0% | +10% | +20% | +30% | +40% | 8.82 |
| ***Jojemnen*** | -40% | -30% | -20% | -10% | 0% | +10% | +20% | +30% | +40% | 2.64 |
| ***Beaulieaux*** | -40% | -30% | -20% | -10% | 0% | +10% | +20% | +30% | +40% | 0.80 |
| ***Slingerman*** | -40% | -30% | -20% | -10% | 0% | +10% | +20% | +30% | +40% | 0.58 |

Figure 3: Decision screen D, Experiment 2

| Share | Estimated Performance over next 12 months | | | | | | | | | Company Identifier Code |
|---|---|---|---|---|---|---|---|---|---|---|
| *Undersill* | -40% | -30% | -20% | -10% | 0% | +10% | +20% | +30% | +40% | 37927914 |
| *Ightsbry* | -40% | -30% | -20% | -10% | 0% | +10% | +20% | +30% | +40% | 79059225 |
| *Jillman* | -40% | -30% | -20% | -10% | 0% | +10% | +20% | +30% | +40% | 66832069 |
| *Foleman* | -40% | -30% | -20% | -10% | 0% | +10% | +20% | +30% | +40% | 33128338 |
| *Maerst* | -40% | -30% | -20% | -10% | 0% | +10% | +20% | +30% | +40% | 35081337 |
| *Mayville* | -40% | -30% | -20% | -10% | 0% | +10% | +20% | +30% | +40% | 24767569 |
| *Creaumy* | -40% | -30% | -20% | -10% | 0% | +10% | +20% | +30% | +40% | 99648795 |

Figure 4: Decision screen E, Experiment 2

## 3 Further analyses

In this section of the appendix, we present the results discussed in Section ??. In the second experiment, we asked participants whether they had any prior investment experience, and whether they believe that the fluency effect exists.[1]

Figure 6 and Table 1 display results for the separation by investment experience. All main effects are confirmed for both groups, and there are no significant differences between return expectations of participants with and without investment experience. Figure 7 and Table 2 show the same results, but separated by whether participants believe in the fluency effect or not.

**Table 1**
Effect of prior investment experience

| Treatment | % participants | return expectation (investors) | return expectation (non-inv.) | $t$ | df | $p$ |
|---|---|---|---|---|---|---|
| 2A | 53 | 28.65 | 27.29 | -0.44 | 104.66 | 0.664 |
| 2B | 50 | 12.41 | 12.49 | 0.03 | 76.06 | 0.980 |
| 2C | 45 | 11.94 | 11.92 | -0.01 | 108.39 | 0.993 |
| 2D | 42 | 4.11 | 4.90 | 0.30 | 80.81 | 0.765 |
| 2E | 39 | 2.29 | 0.39 | -1.13 | 82.89 | 0.260 |

The table reports the proportion of participants who state that they have prior investment experience, average return expectations by whether they have experience or not, and test statistics for pairwise comparisons between groups, all by treatment.

[1] Specifically, we asked if they believe 'that the ease by which you can pronounce a company's name provides a signal for its future performance in real world financial markets?'.

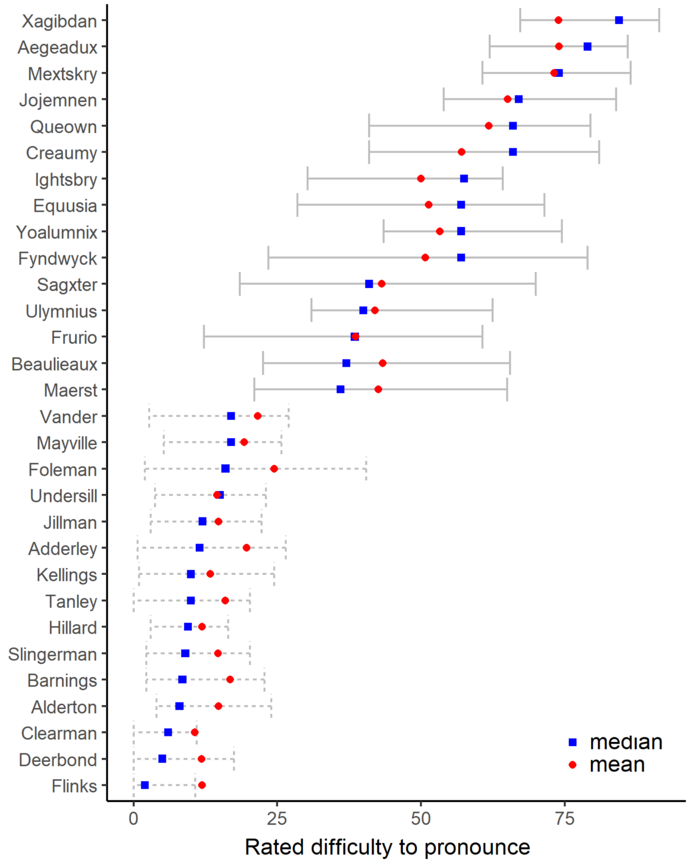


Figure 5: This figure shows mean and median scores and 95% confidence intervals for the fluency scores assigned by the participants to the 30 companies in this study on a rating scale from 0–100. The upper half represents non-fluent company names, the lower half fluent company names.

**Table 2**
Effect of belief in real-world fluency effect

| Treatment | % believers | return expectation (believers) | return expectation (non-bel.) | $t$ | df | $p$ |
|---|---|---|---|---|---|---|
| 2A | 60 | 30.24 | 21.98 | -2.43 | 65.52 | 0.018 |
| 2B | 60 | 14.00 | 10.16 | -1.11 | 62.81 | 0.273 |
| 2C | 66 | 15.31 | 5.26 | -4.08 | 48.67 | $< 0.01$ |
| 2D | 56 | 6.89 | 1.70 | -1.99 | 73.95 | 0.050 |
| 2E | 61 | 1.89 | 0.59 | -0.82 | 94.49 | 0.413 |

The table reports the proportion of participants who state that they believe in the fluency effect, average return expectations by whether they believe in the fluency effect or not, and test statistics for pairwise comparisons between groups, all by treatment.

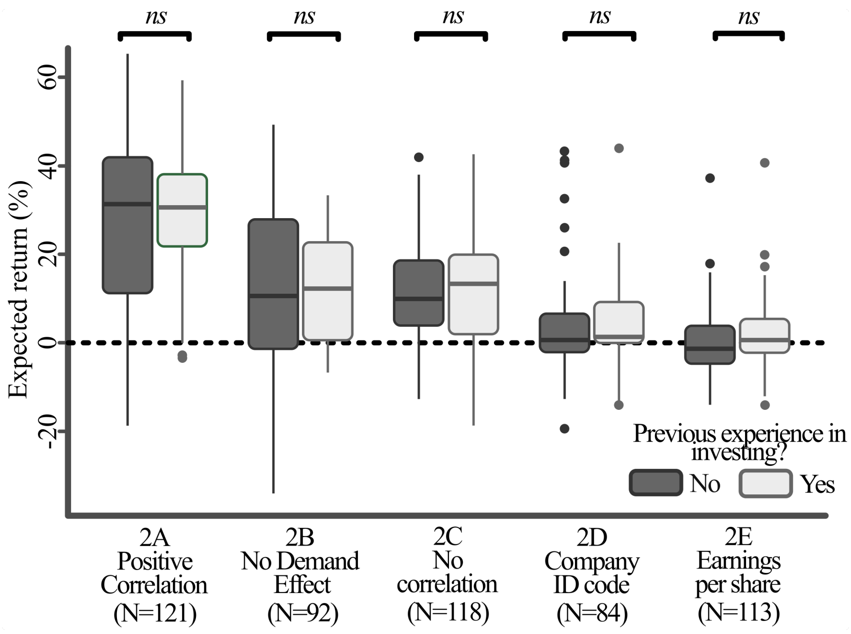


Figure 6: Average return expectations for fluent and non-fluent company names, by treatment and separated by whether participants have any prior investment experience. Significance based on between-subject $t$-tests.

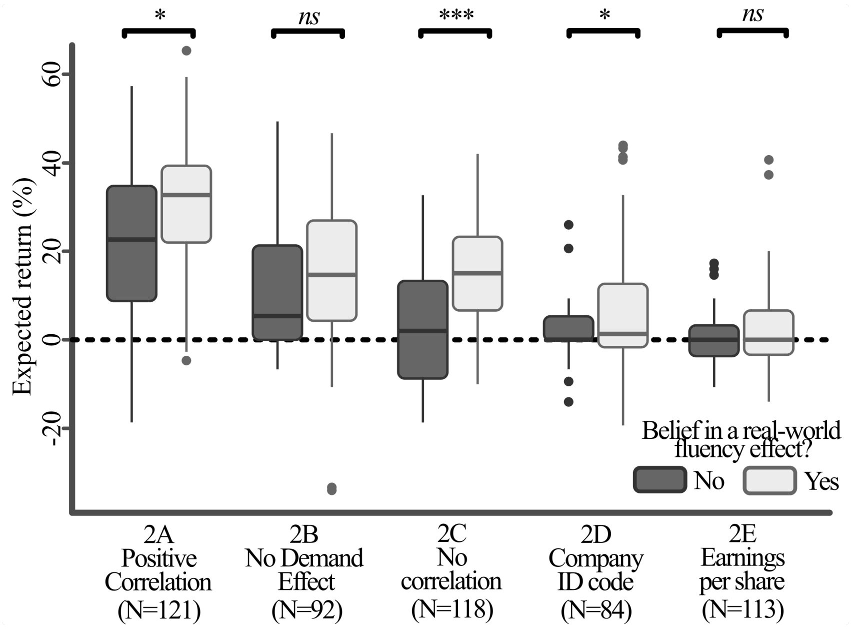


Figure 7: Average return expectations for fluent and non-fluent company names, by treatment and separated by whether participants believe in the fluency effect or not. Significance based on between-subject *t*-tests.
